# Supplementary material for: HIVprotI: an integrated web based platform for prediction and design of HIV proteins inhibitors
Source: J Cheminform. 2018 Mar 9;10:12. doi: 10.1186/s13321-018-0266-y (PMC5845081; doi:10.1186/s13321-018-0266-y)
Supplement: Supplementary file 2 — Additional file 2. Source code of HIVProtI web server. [file 13321_2018_266_MOESM2_ESM.zip › HIVprotI_Source-code/hivprotI/weka-3-6-6/doc/weka/core/neighboursearch/package-summary.html]

weka.core.neighboursearch


---


|  |  |  |
| --- | --- | --- |
| **Overview** \| **Package** \| Class \| **Tree** \| **Deprecated** \| **Index** \| **Help** \| **Weka's home** | |  |
| **PREV PACKAGE**   **NEXT PACKAGE** | **FRAMES**    **NO FRAMES**     **All Classes** |  |


---

## Package weka.core.neighboursearch

| **Class Summary** | |
| --- | --- |
| **BallTree** | Class implementing the BallTree/Metric Tree algorithm for nearest neighbour search.  The connection to dataset is only a reference. |
| **CoverTree** | Class implementing the CoverTree datastructure.  The class is very much a translation of the c source code made available by the authors.    For more information and original source code see:    Alina Beygelzimer, Sham Kakade, John Langford: Cover trees for nearest neighbor. |
| **KDTree** | Class implementing the KDTree search algorithm for nearest neighbour search.  The connection to dataset is only a reference. |
| **LinearNNSearch** | Class implementing the brute force search algorithm for nearest neighbour search. |
| **NearestNeighbourSearch** | Abstract class for nearest neighbour search. |
| **PerformanceStats** | The class that measures the performance of a nearest neighbour search (NNS) algorithm. |
| **TreePerformanceStats** | The class that measures the performance of a tree based nearest neighbour search algorithm. |

---


|  |  |  |
| --- | --- | --- |
| **Overview** \| **Package** \| Class \| **Tree** \| **Deprecated** \| **Index** \| **Help** \| **Weka's home** | |  |
| **PREV PACKAGE**   **NEXT PACKAGE** | **FRAMES**    **NO FRAMES**     **All Classes** |  |


---
